# Orthotopic Kidney Transplantation in Mice: Technique Using Cuff for Renal Vein Anastomosis

**DOI:** 10.1371/journal.pone.0077278

**Published:** 2013-10-14

**Authors:** Hao Chen, Ying Zhang, Donghang Zheng, Raaj Kumar Praseedom, Jiahong Dong

**Affiliations:** 1 Hospital & Institute of Hepatobiliary Surgery, Chinese PLA General Hospital, Beijing, China; 2 Department of Cardiology, College of Medicine, University of Florida, Gainesville, Florida, United States of America; 3 Key Laboratory of Combined Multiorgan Transplantation, First Affiliated Hospital, College of Medicine, Zhejiang University, Hangzhou, Zhejiang, China; 4 Department of Microbiology, Chinese PLA General Hospital, Beijing, China; 5 Department of HPB-Transplant Surgery, NIHR Comprehensive Biomedical Campus, Addenbrookes Hospital, Cambridge, United Kingdom; Bascom Palmer Eye Institute, University of Miami School of Medicine, United States of America

## Abstract

Mouse renal transplantation is a technically challenging procedure. Although the first kidney transplants in mice were performed over 34 years ago and refined some years later, the classical techniques of mouse renal transplantation required clamping both vena cava and aorta simultaneously and carry out suture anastomoses of the renal artery and vein in a heterotopic position. In our laboratory, we have successfully developed mouse orthotopic kidney transplantation for the first time, using a rapid “cuffed” renal vein technique for vessel anastomosis, wherein the donor’s renal vein was inserted through an intravenous catheter, folded back and tied. During grafting, the cuffed renal vein was directly inserted into the recipient’s renal vein without the need for the clamping vena cava and suturing of renal vein. This technique allowed for the exact transplantation of the kidney into the original position, compared to the classical technique, and has significantly shortened the clamping time due to a quicker and precise anastomosis of renal vein as described. This also allowed for a quicker recovery of the lower extremity activity, reduction in myoglobinuria with resultant kidney graft survival of 88.9%. Thus we believe that the cuffed renal vein technique simplifies microvascular anastomoses and affords important additional benefits.

## Introduction

Murine kidney transplantation has gained widespread use as genetically modified strains can be used to study the molecular mechanisms of renal allograft injury [1,2]. However, due to the technical complexity and high mortality rates, few laboratories in the world have mastered this procedure. 

Skoskiewicz et al reported the first mouse kidney transplantation in 1973 [3], which was later improved by Zhang and Han [4,5], and recently modified by Song Rong [6]. Generally, this classical technique required cross-clamping proximally and distally a section of aorta and inferior vena cava (IVC) with two microvascular clamps, elliptical arteriotomy and longitudinal venotomy , end-to-side anastomosis of the donor renal vessels to the aorta and IVC below the level of the native renal vessels. This technique has the disadvantage of aortic and vena caval cross clamping which prolongs the ischemia time of lower extremities and subsequently inducing rhabdomyolysis and worsening renal function. 

In the present study we describe a new method of orthotopic transplantation where the renal vein anastomosis is carried out using a cuffed technique thereby reducing the aortic cross clamp time. We also compared the outcomes of both techniques (the cuffed renal vein and the classical technique) in terms of time taken for arterial and venous anastomoses, aortic and vena caval cross clamp times, recovery time for lower limb activity and urinary myoglobin. In addition, long-term survival and renal function with the new technique were monitored over 10 weeks.

## Materials and Methods

### Animals

 Male C57Bl/6 mice weighing 20-28g, from 8 to 10 weeks of age were obtained from the Jackson Lab and housed at the Animal Care Services (ACS), University of Florida. Male C57Bl/6 mice were randomly used as donors and recipients. All animals were held under standard conditions at constant temperature, humidity, and light/dark cycles. They were fed with a standard diet and had free access to tap water. The protocol of animal experiments was approved by the Institutional Ethical Committee of Animal Experimentation of the University of Florida, and the experiments were performed strictly according to governmental and international guidelines on animal experimentation. Animals were treated according to requirements for Biosafety and Animal Ethics which requires ‘Units and individuals who are conducting the production and use of experimental animal production, should treat animals humanely and protect animal welfare, should not tease and abuse animals. The use of experimental animals should be in accordance to the scientific, rational and humane requirements. It is advised and encouraged to minimize the use of laboratory animals to reduce suffering of animals to be disposed of, and to explore of alternative methods in replacing animal testing and use. Every effort was made to minimize any suffering of the animals used in this study. All animals received care in compliance with the Principles of Laboratory Animal Care. The experimental protocol was approved by the local Animal Care and Research Committee (IACUC). 

### Experimental Design

 Both techniques (the classical and the cuffed renal vein) were utilized in isogenic transplantations. With the classical technique 20 isogenic kidney transplantations were performed. With the new cuffed renal vein technique, 30 isogenic kidney transplantations were performed. With both techniques, the recipient underwent contralateral native nephrectomy 5 days following the transplant procedure. The procedure was considered a technical success if the recipient survived more than 4 days following contralateral native nephrectomy. An autopsy was carried out if the recipient died prematurely. In addition, 18 long-term experiments with the cuffed renal vein were performed for 10 weeks with monitoring of survival and renal function.

### Surgical Technique

 Donor Organ Harvest: The donor mouse is anaesthetized with an intramuscular injection of mixture of ketamine (100 mg/kg) and xylazine (5 mg/kg). A midline incision is made from the sternum to the pubis. The left kidney is exposed by moving the intestines to the right side and retracting the stomach with a mosquito clamp. The left kidney is isolated by ligating and dividing the adrenal and testicular vessels with 9-0 silk suture. The aorta and inferior vena cava (IVC) is dissected at their junction with the left renal artery and vein by ligating and dividing a few lumbar branches. The infrarenal aorta is ligated with a 9-0 silk suture to minimize bleeding. The left ureter is dissected from the bladder to renal hilum. The ureterovesical junction is exposed using caudal retraction on the bladder dome. A small, elliptical patch of bladder containing the left ureterovesical junction is excised and used for urinary reconstruction in the recipient. The IVC is ligated below the renal artery and vein with a 9-0 silk suture. The supra renal aorta is ligated and introduce a 28-gauge ½ needle into the infra renal aorta for perfusion. The graft is then perfused slowly and evenly in situ with 0.5 mL of cold, heparinized (100unit/ml), lactated Ringer’s solution for 45 seconds. The supra renal IVC is ligated with a 9-0 silk suture. The renal vein at its junction with the IVC is divided leaving sufficient length for cuff preparation. The aorta is divided obliquely, approximately 2mm below the renal artery. The kidney and associated vessels are then completely freed by cauterizing the tissue surrounding the major vessels, and then removed and stored in saline at 4°C for further preparation. The donor mouse is euthanized by cervical dislocation under anesthesia.

**Figure 1:**
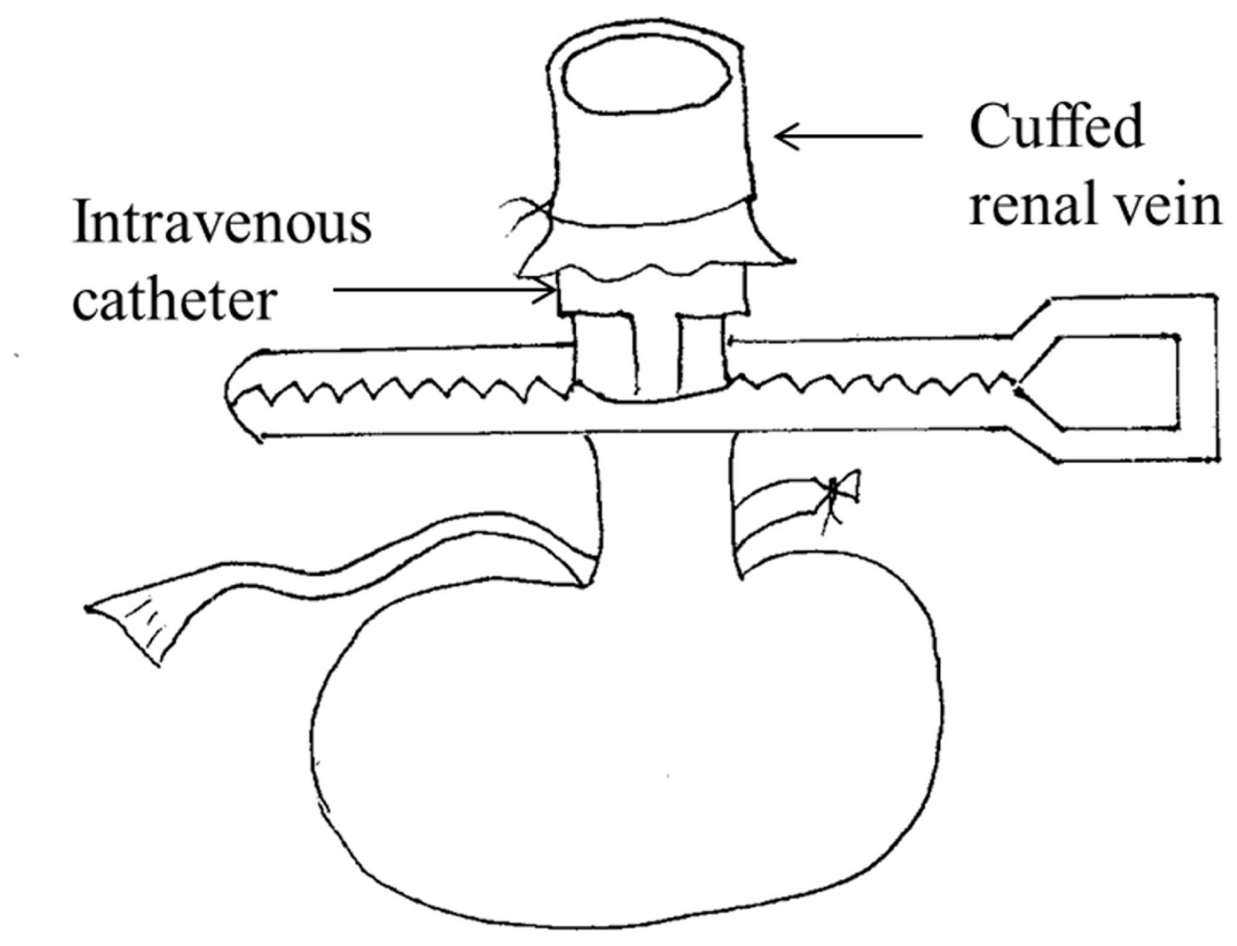
The cuffed renal vein technique. The renal vein was cleaned for insertion through 22G intravenous catheter (outer diameter 1.1 mm), using a cuff length of about 0.7-0.9 mm and an equal vein length, which was folded back and tied.

 Donor renal vein cuffing: The renal vein is cleaned for insertion through 22G intravenous catheter (outer diameter 1.1 mm, inter diameter 0.9mm), using a cuff length of about 0.7-0.9 mm and an equal vein length, which is folded back and tied (Figure 1). These steps are facilitated by a stabilizing clamp.

**Figure 2:**
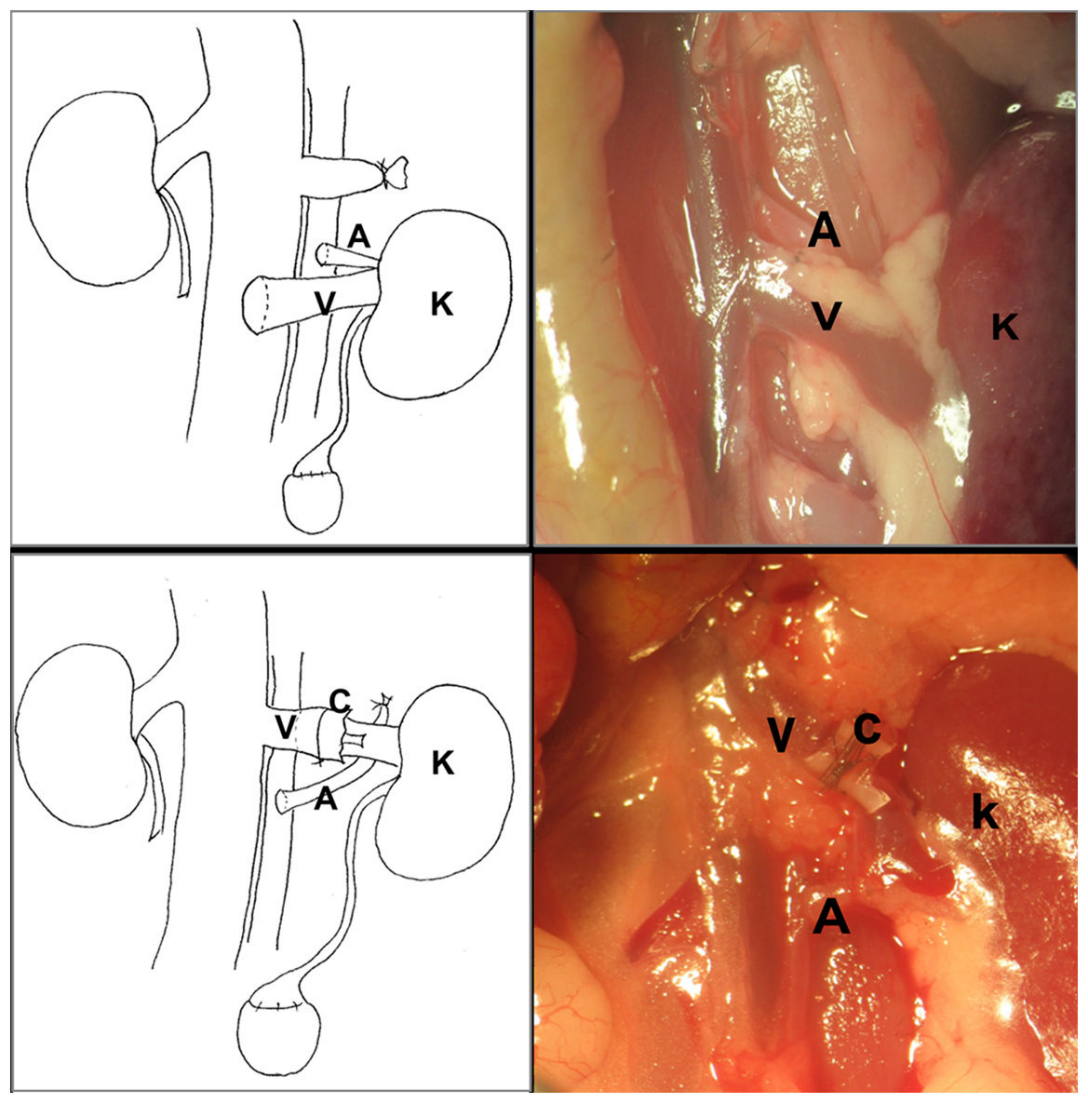
Orthotopic Kidney Transplantation in the Mice Using Cuff for Renal VeinAnastomosis. Top panel shows a classical kidney transplantation, lower panel illustrates the cuffed renal vein techniques. Left portrait is match with the right in vivo picture. A-aorta, V-vein, K- kidney, C-cuff.

 Recipient Operation: After anesthesia the abdomen is opened via midline incision, and the bowel was moved to the right abdomen and covered with moist gauze. The left renal artery and ureter are isolated. The left renal vein is isolated and clamped with a microvascular clamp (world precision instruments, Inc. straight 0.75 x 4mm jaw, item NO 501779-G) at its junction with the IVC. The renal artery, vein and the ureter are cut at the renal hilum and the kidney is removed The donor kidney is removed from ice, placed intra-abdominally in the left flank and kept moist with a gauze constantly soaked in cold saline . Now, the graft renal vein cuff is inserted into the recipient renal vein which is kept under tension with a clamp and inflated with injected saline. A silk tie is placed to secure the vein over the cuff. The infrarenal aorta is isolated and cross-clamp after ligating the lumbar branches. An 11-0 nylon suture is placed through the full thickness of aorta and retracted in order to make an elliptical arteriotomy by a single cut (about one-fifth of the diameter of the vessel). The aorta is irrigated with heparinized saline to clear intraluminal blood or clots. Two stay sutures are placed at the proximal and distal apex of the recipient’s arteriotomy. After ensuring that the orientation of the donor renal artery, an end-to-side anastomosis is performed between the donor aorta and recipient aorta using interrupted 11-0 nylon sutures. Only three or four sutures are required for each side. The kidney is then reperfused by removing the aortic and renal vein clamps. Gentle pressure is applied to the anastomotic site with a dry cotton swab for 15 seconds after perfusion. A cystotomy is made in the dome of the bladder and two stay sutures of 9-0 nylon are placed to anastomose the donor’s small bladder patch with the recipient’s bladder dome after ensuring correct orientation of the donor ureter. Each side of the anastomosis is sutured with four or five interrupted 9-0 sutures. The abdomen is closed in two layers with continuous 5-0 sterile nonabsorbable suture.(Figure 2)

### Post-operative Care of Recipient

 After closure of body wall and skin, 0.3 mL NS is administered subcutaneously, In addition, recipients receive 0.05 mg/kg buprenorphine intramuscularly on day 0 and day 1 post surgery for pain control. For the first 24 hours postoperatively the mouse is placed on an electric warming blanket until fully awake. Skin sutures are removed 2 weeks after the surgery.

### Recipient Nephrectomy

 The recipient is anaesthetized 5 days post kidney transplantation. The abdomen is opened through a midline incision. The intestine is covered with saline-soaked gauze and carefully retracted it to the left side. The right kidney is removed after ligating the right ureter, renal vein and renal artery. The abdomen is closed in two layers with continuous 5-0 sterile non-absorbable sutures.

#### Protein level in urine

 Urine was obtained with bladder puncture at 2 hours post operatively and the protein levels in urine was tested by the SIEMENS multistix® 10 SG. We used this as a surrogate marker for renal injury and dysfunction secondary to myoglobinuria, which is normally excreted rapidly within 2 hours in the mice.

#### Kidney transplant function

 Renal function was determined by collecting retro-orbital blood at the indicated time points. Serum creatinine levels were measured using LC-MS/MS.

#### Evaluation Parameters

 Times for arterial, venous anastomosis, lower extremity reactivity were measured for analysis. Because of the ischemia and reperfusion, mice cannot move their legs from major surgery and anesthesia, in order to reliably evaluate the leg movements without observer bias, the lower extremity activity recovery was defined as starting shivering of both leg after renal transplantation.

### Statistical Analysis

 All data are presented as mean±SD. Statistical analysis was performed by the t test and Kruskal–Wallis test using SPSS 13.0 software. Success rate was analyzed by X^2^test. Survival rates were assessed by the Kaplan–Meier method. The log-rank test was used to compare significance. A significance level of P<0.05 was considered as sufficient in all experimental groups.

## Results

### The cuffed renal vein technique significantly shortened the operation time

**Figure 3:**
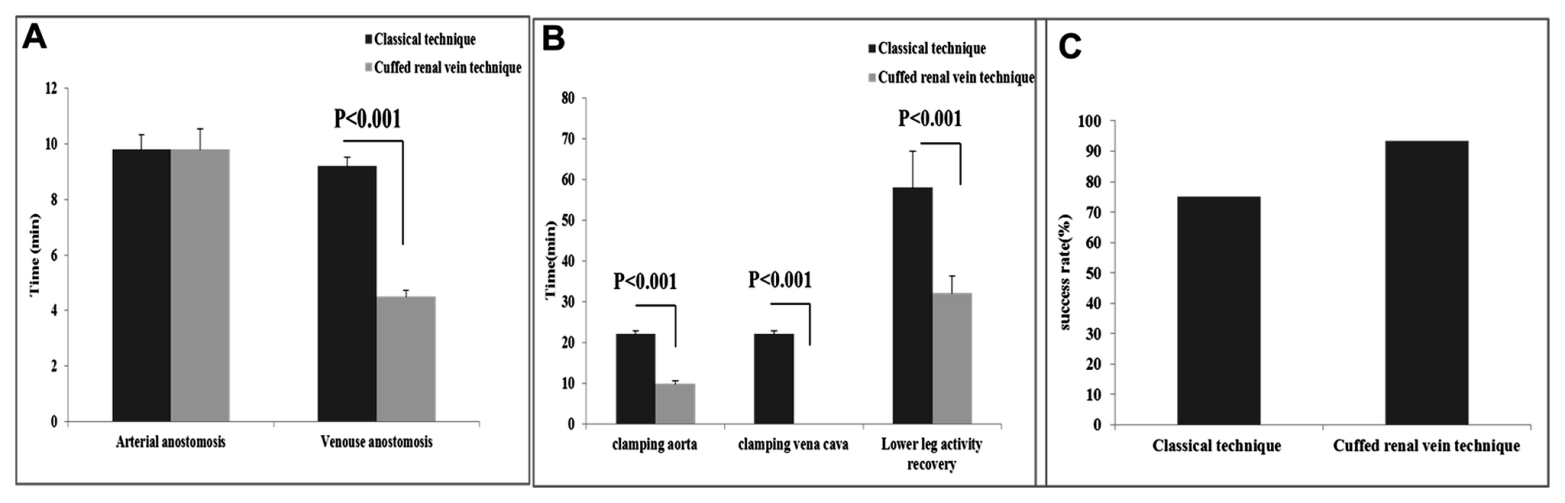
The cuffed renal vein technique significantly shortened the operation time. **A**) Total operative time was reduced with the new cuffed renal vein technique, as evidenced by the significantly reduced times required to carry out the renal anastomoses (p<0.001,). **B**) The clamping times for vena cava and aorta were dramatically reduced in cuffed renal vein technique compared to the classical technique (p<0.001), and consequently the recovery of lower limb activity was significantly quicker with cuffed renal vein technique (p<0.001). **C**) The survival improved significantly from 75% with the classical technique to 93.3% with the cuffed renal vein technique (P < 0.001).

 Overall operative time, aortic cross clamp time and lower legs activity recovery time were compared between the classical technique (n=20) and the new cuffed renal vein technique (n = 30). The overall operative time was reduced with the new cuffed renal vein technique, as evidenced by the significantly reduced times required to prepare the renal anastomoses (p<0.001, Figure **3A**). In this model, instead of blocking both vena cava and aorta before anastomosis in classical technique, only aorta was blocked, the clamping times for vena cava and aorta were dramatically reduced in cuffed renal vein technique comparing to the classical technique (p<0.001, Figure **3B**), and consequently recovery of lower extremity activity was significantly quicker with cuffed renal vein technique than that with classical technique (p<0.001, Figure **3B**). Additionally, the technical success rate was significantly better from 75% with the classical technique to 93.3% with the cuffed renal vein technique (P < 0.001, Figure **3C**). In “classical technique group” 2 animals were dead of aorta thrombosis and fat embolism respectively, 2 animals were dead of anastomotic stenosis of renal vein, one animal was dead of abdominal infection from perforation of colon. In the "experimental cuff technique group", one animal was dead of aorta thrombosis, the other one was dead of aorta bleeding. The cold ischemia times were 22.6±2.1 and 22.9± 1.9 minutes in classical kidney transplantation and the cuffed renal vein techniques respectively, the warm ischemia time were 19.0±2.5 and 14.4±2.8 minutes respectively.

### The cuffed renal vein technique significantly reduced the level of urinary protein

**Figure 4:**
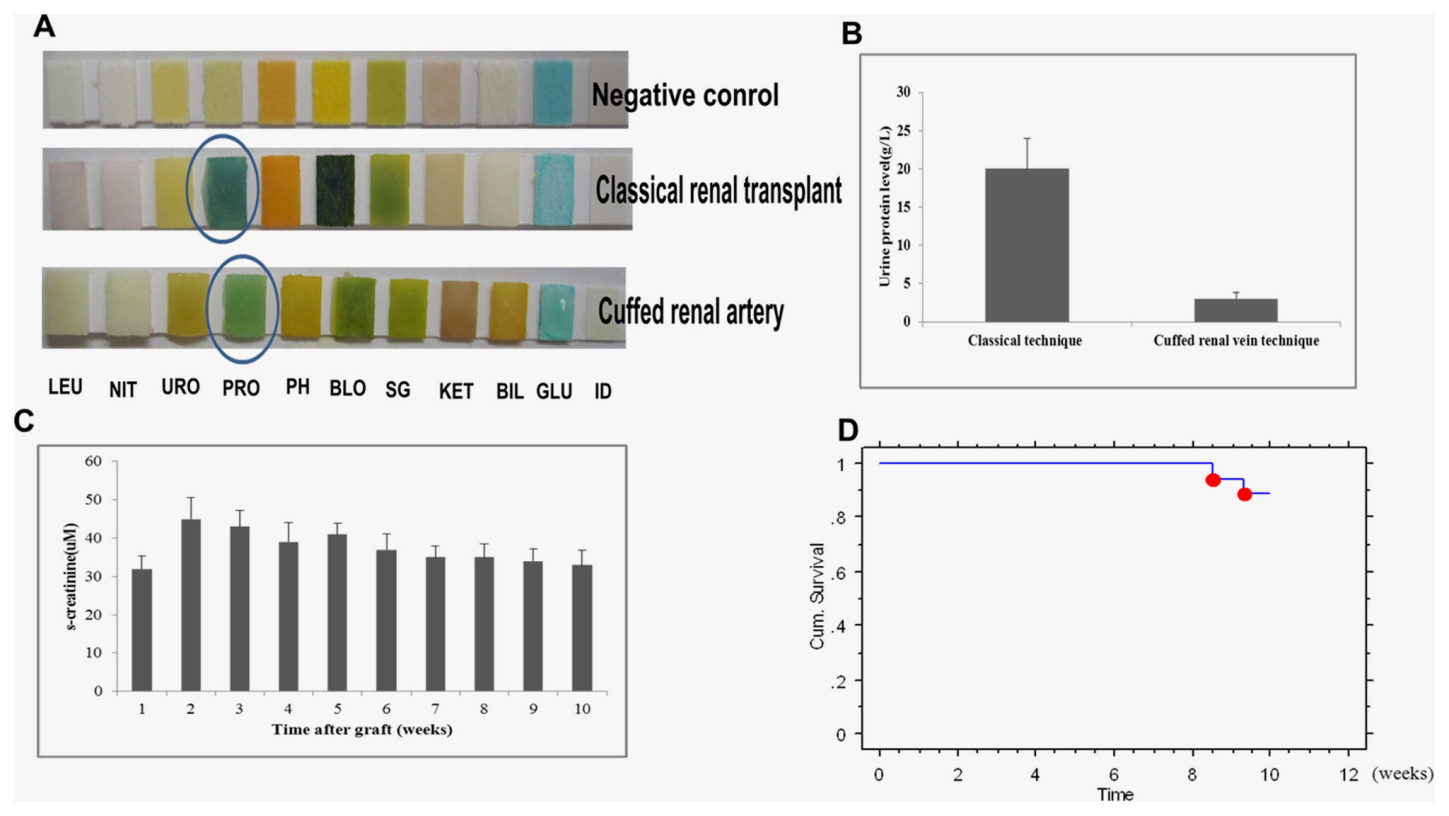
The cuffed renal vein technique significantly improves the renal function. **A**,**B**) The level of urinary protein in mice with cuffed renal technique was significantly lower than in mice operated using classical technique (p<0.05). **C**, **D**) Long-term survival over 10 weeks with the cuffed renal vein technique was 88.9%, Serum creatinine increased slightly but not significantly after transplantation and remained stable during the observation period of 10 weeks.

 Since myoglobin can be released from damaged muscle and rapidly excreted in the urine, two hour postoperatively, the urine was achieved by bladder puncture and tested for urinary protein level, we found that the level of urinary protein in mice with the cuffed renal vein technique was significantly lower than in mice operated using the classical technique (p<0.05, Figure **4A,B**)

The cuffed renal vein techniques significantly improved the renal function and long-term survival.

 In addition to the short-term outcome, renal function and long-term survival were studied in isogenic kidney transplant recipients. Long-term survival over 10 weeks with the cuffed renal vein technique was 88.9%, Serum creatinine increased slightly but not significantly after transplantation and remained stable during the observation period of 10 weeks (Figure **4C,D**).

## Discussion

 The kidney transplant model offers an opportunity to explore a life-supporting model following bilateral nephrectomy of the native kidneys of the recipient [7], especially important for the investigation of the role of certain genes in transplant rejection by studying knockout, transgenic, congenic, and inbred mouse strains and can be used to test new experimental therapeutic strategies.

 However, mouse kidney transplantation is a very demanding microsurgical procedure, which requires long intense training and meticulous technique [8]. The murine kidney is extremely sensitive to ischemia/reperfusion injury, and to perform a successful operation the secondary warm ischemic time should be less than 35 minutes [9,10]. As described by Martins, kidney transplantation in these settings is challenging, and the success rates of even experienced surgeons vary between 40 and 70% [11,12]. Zhang and coworkers have reported the highest survival rates in the literature ranging between 79 and 90% [13].

 Technical failure during surgery, most commonly presenting as bleeding or stenosis at the sites of vessel anastomosis are still major obstacles to long-term success after mouse kidney transplantation. Anastomotic stenosis, bleeding, and thrombosis are severe complications causing subsequent graft failure secondary to technical problems [9]. Therefore, the construction of high quality and reproducible anastomoses plays a key role in achieving good graft survival and therefore further experimental results. We developed a new modified technique in order to simplify the procedure. The new cuffed renal vein method offers a rapid easily performed and reproducible technique. Ligation by a single tie outside the renal vein after insertion of the cuffed renal vein of donor into the renal vein of recipient avoids the need for a sutured anastomosis and thereby potentially reducing the usual anastomotic complications such as bleeding and thrombosis. Additionally the intravenous catheter cuffed by renal vein acts as a scaffold to prevent stenosis of the anastomosis site. Consequently, our modified cuffed procedure can effectively eliminate the three major complications associated with this animal model of renal transplantation: anastomotic bleeding and stenosis and thrombosis from renal vein.

 Ischemia time is one of the important factors impacting transplantation results [14,15]. Ischemia itself can cause severe damage because of the potential for a build-up of metabolic wastes. Reperfusion after a period of ischemia can actually be more harmful than the ischemia itself. Ischemia-reperfusion results in vascular edema and leukocytes building up in small capillaries which accelerates the process of thrombosis and renal failure after transplantation. With the cuffed renal vein technique, the ischemia time was significantly reduced compared to the classical technique. The duration of the warm ischemic time contributes to acute renal failure [16], rhabodomyolysis of the lower extremity [17], necrosis of ureter and bladder after transplantation. Using the cuffed renal vein method there was a significant reduction in the warm ischemia time, We believe that this could potentially promote the quicker recovery of graft by reducing apoptosis, reducing the formation of ROS (reactive oxygen specie), reducing the production of myoglobin, Similarly a quicker recovery of lower limb activity in this study suggests an improved milieu interior in the transplant recipient and decreased the ureteral ischemia and subsequent ureteral necrosis and leakage contribute to long-term higher survival rate.

 In conclusion, we have shown that by using the cuffed renal vein technique there were significant reductions in anastomosis time, cross clamp time and warm ischemia time associated with an improved recovery and survival 

 To the author’s knowledge this is the first report in the mouse using intravenous catheter cuffed renal vein technique. The potential advantages of this technique applied in the murine model may be confirmed in the long-term case-control studies.
